# December’s Garnering

**Published:** 1902-01

**Authors:** 


### "DECEMBER'S GARNERING."

THE ranks of the profession of the East were severely stricken in December. Baltimore was first deprived of one of her most popular members of the profession, and one endeared to all who knew him—Dr. Thomas F. Barron; Philadelphia will miss in the death of Dr. James Beatty one of the most genial members of the younger generation in the profession; while in the death of the senior editor of this JOURNAL, Dr. Rush Shippen Huidekoper, the profession at home and abroad has lost one of its most valued leaders, and those who knew him one of the most companionable of men, and, in the language of Senator Carter, "a prince of good fellows." Surely our cup of sorrow in the closing days of the old year was filled to overflowing. While gone from our midst, their memories and good deeds will live long after them.

---

THE family of our late editor, Dr. R. S. Huidekoper, beg leave to express through the JOURNAL columns their deep and sincere appreciation of the many expressions of sympathy and appreciation of our esteemed colleague.

